# Supplementary material for: Voluntary Running Improves Behavioral and Structural Abnormalities in a Mouse Model of CDKL5 Deficiency Disorder
Source: Biomolecules. 2023 Sep 15;13(9):1396. doi: 10.3390/biom13091396 (PMC10527551; doi:10.3390/biom13091396)
Supplement: Supplementary file 1 [file biomolecules-13-01396-s001.zip › Figure S1.pdf]

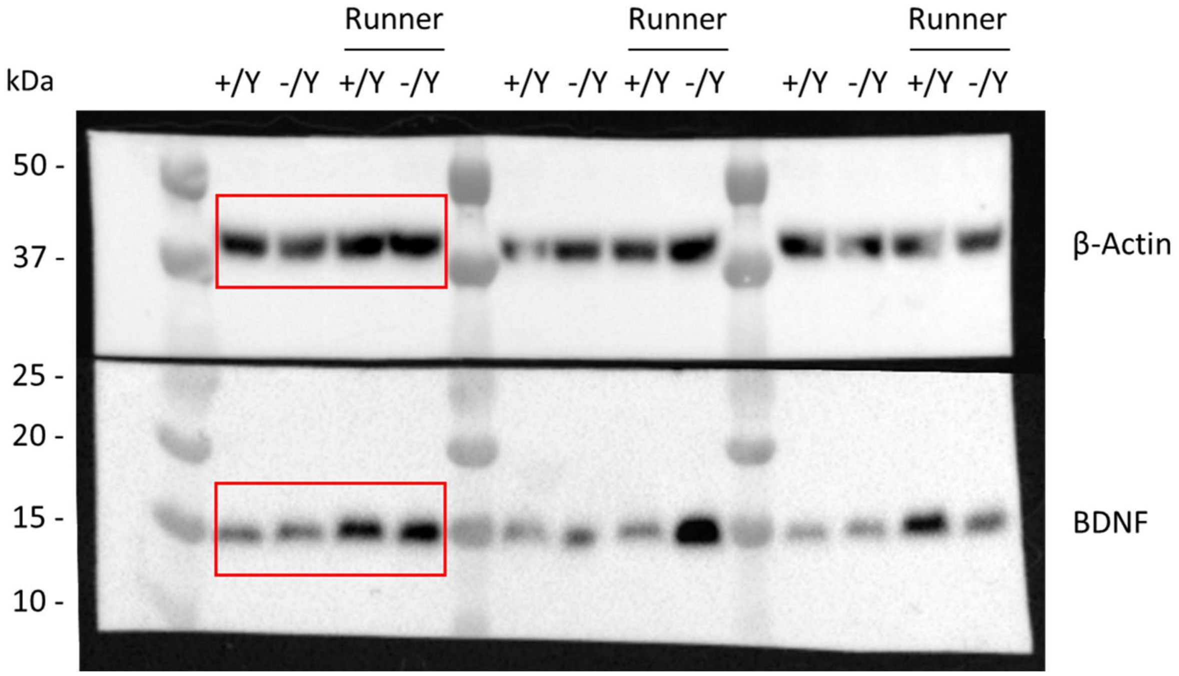

Figure S1. Image of the parts of the membrane, between 50 and 25 kDa and 25 and 10 kDa, separately incubated with the anti-β-Actin and the anti-BDNF antibodies. The red boxes indicate the parts of the image shown in Figure 6D.
