# Supplementary material for: Voluntary Running Improves Behavioral and Structural Abnormalities in a Mouse Model of CDKL5 Deficiency Disorder
Source: Biomolecules. 2023 Sep 15;13(9):1396. doi: 10.3390/biom13091396 (PMC10527551; doi:10.3390/biom13091396)
Supplement: Supplementary file 1 [file biomolecules-13-01396-s001.zip › Table S1.pdf]

				Behavioral testing			
Cohort	Sex	Genotype	Treatment	Hind-Limb Clasping	Object Exploration	Passive Avoidance	Body weight
1	M	+/Y	NO RUN	✓	-	✓	-
1	M	+/Y	NO RUN	✓	-	✓	-
1	M	+/Y	NO RUN	✓	-	✓	-
1	M	+/Y	NO RUN	✓	-	✓	✓
1	M	+/Y	NO RUN	✓	-	✓	✓
1	M	+/Y	NO RUN	✓	-	✓	✓
1	M	+/Y	NO RUN	✓	-	✓	✓
1	M	+/Y	NO RUN	✓	-	✓	✓
2	M	+/Y	NO RUN	✓	✓	✓	✓
2	M	+/Y	NO RUN	✓	✓	✓	✓
2	M	+/Y	NO RUN	✓	✓	✓	✓
3	M	+/Y	NO RUN	-	✓	✓	-
3	M	+/Y	NO RUN	-	✓Outlier	✓	-
3	M	+/Y	NO RUN	-	✓	✓	-
3	M	+/Y	NO RUN	-	✓	✓	-
4	M	+/Y	NO RUN	✓	✓	✓	✓
4	M	+/Y	NO RUN	✓	✓	✓	✓
4	M	+/Y	NO RUN	✓	✓	✓	✓
5	M	+/Y	NO RUN	✓	✓	✓	✓
5	M	+/Y	NO RUN	✓	✓	✓	✓
5	M	+/Y	NO RUN	✓	✓	✓	✓
5	M	+/Y	NO RUN	✓	✓	✓	✓
1	M	-/Y	NO RUN	✓	-	✓	✓
1	M	-/Y	NO RUN	✓	-	✓	✓
1	M	-/Y	NO RUN	✓	-	✓	✓
1	M	-/Y	NO RUN	✓	-	✓	✓
2	M	-/Y	NO RUN	✓	✓	✓	✓
3	M	-/Y	NO RUN	-	✓	✓	-
3	M	-/Y	NO RUN	-	✓	✓	-
3	M	-/Y	NO RUN	-	✓	✓	-
3	M	-/Y	NO RUN	-	✓	✓	-
3	M	-/Y	NO RUN	-	✓	✓	-
3	M	-/Y	NO RUN	-	✓	✓	-
4	M	-/Y	NO RUN	✓	✓Outlier	✓	✓
4	M	-/Y	NO RUN	✓	✓	✓	✓
4	M	-/Y	NO RUN	✓	✓	✓	✓
6	M	-/Y	NO RUN	✓	✓	✓	✓
6	M	-/Y	NO RUN	✓	✓	✓	✓
6	M	-/Y	NO RUN	✓	✓	✓	✓
4	M	+/Y	RUN	✓	✓	✓	✓
4	M	+/Y	RUN	✓	✓	✓	✓
4	M	+/Y	RUN	✓	✓	✓	✓
4	M	+/Y	RUN	✓	✓	✓	✓
5	M	+/Y	RUN	✓	✓	✓	✓
5	M	+/Y	RUN	✓	✓	✓Outlier	✓
5	M	+/Y	RUN	✓	✓	✓	✓
5	M	+/Y	RUN	✓Outlier	✓	✓	✓
1	M	-/Y	RUN	✓	-	-	✓
1	M	-/Y	RUN	✓	-	-	✓
1	M	-/Y	RUN	✓	-	-	✓
1	M	-/Y	RUN	✓	-	✓	✓
2	M	-/Y	RUN	✓	✓	✓	✓
2	M	-/Y	RUN	✓	✓	✓	✓
2	M	-/Y	RUN	✓	✓Outlier	✓	✓
2	M	-/Y	RUN	✓	✓	✓	✓
6	M	-/Y	RUN	✓	✓	✓	✓
6	M	-/Y	RUN	✓	✓	✓	✓
6	M	-/Y	RUN	✓	✓	✓	✓
6	M	-/Y	RUN	✓	✓	✓	✓

Table S1. Mouse cohorts used for behavioral tests. For each mouse cohort (1-6) used in the present study: genotype (wild type: +/Y or *Cdkl5* KO: -/Y), type of treatment (sedentary: NO RUN or runner: RUN) and type of behavioral test (performed: ✓ or not performed: -) are reported.
