# Supplementary material for: Voluntary Running Improves Behavioral and Structural Abnormalities in a Mouse Model of CDKL5 Deficiency Disorder
Source: Biomolecules. 2023 Sep 15;13(9):1396. doi: 10.3390/biom13091396 (PMC10527551; doi:10.3390/biom13091396)
Supplement: Supplementary file 1 [file biomolecules-13-01396-s001.zip › Table S2.pdf]

**Primary antibodies**				
**Target**	**Description**	**Dilution**	**Manufacturer**	**Catalog number**
AIF-1	Rabbit polyclonal	IHC 1:300	Thermo Fisher Scientific	PA5-21274
DCX	Rabbit polyclonal	IHC 1:300	Thermo Fisher Scientific	48-1200
BrdU	Rat monoclonal	IHC 1:200	Abcam	ab1893
BDNF	Rabbit polyclonal	IHC 1:200	Alomone Labs	ANT-010
BDNF	Rabbit polyclonal	WB 1:500	Santa Cruz Biotechnology	sc-546
β-Actin	Mouse monoclonal	WB 1:5000	Sigma-Aldrich	A5441

**Secondary antibodies**			
**Description**	**Dilution**	**Manufacturer**	**Catalog number**
Donkey anti-Rabbit IgG Cy3-conjugated	IHC 1:200	Jackson ImmunoResearch	711-165-152
Goat anti-Rat IgG Cy3-conjugated	IHC 1:200	Jackson ImmunoResearch	112-165-143
Goat anti-Rabbit IgG HRP-conjugated	WB 1:5000	Jackson ImmunoResearch	111-035-003
Goat anti-Mouse IgG HRP-conjugated	WB 1:5000	Jackson ImmunoResearch	115-035-003

Table S2. List of primary and secondary antibodies. IHC = immunohistochemistry; WB = western blotting.
