# Supplementary material for: Voluntary Running Improves Behavioral and Structural Abnormalities in a Mouse Model of CDKL5 Deficiency Disorder
Source: Biomolecules. 2023 Sep 15;13(9):1396. doi: 10.3390/biom13091396 (PMC10527551; doi:10.3390/biom13091396)
Supplement: Supplementary file 1 [file biomolecules-13-01396-s001.zip › Table S3.pdf]

Two-way RM ANOVA

Figure	Time x Genotype		Time		Genotype		Subject	
1C	F (3, 54) = 8.068	P=0.0002	F (2.054, 36.97) = 30.86	P<0.0001	F (1, 18) = 14.08	P=0.0015	F (18, 54) = 5.946	P<0.0001
1D	F (12, 168) = 1.932	P=0.0337	F (4, 168) = 26.06	P<0.0001	F (3, 42) = 0.2072	P=0.8908	F (42, 168) = 181.1	P<0.0001

Two-way ANOVA

Figure	Running x Genotype		Running		Genotype	
2A	F (1, 36) = 0.7865	P=0.3810	F (1, 36) = 6.342	P=0.0164	F (1, 36) = 11.22	P=0.0019
2B	F (1, 36) = 1.557	P=0.2202	F (1, 36) = 10.43	P=0.0027	F (1, 36) = 14.81	P=0.0005
2C	F (1, 36) = 5.755	P=0.0217	F (1, 36) = 1.626	P=0.2104	F (1, 36) = 3.457	P=0.0712
2D	F (1, 36) = 0.01357	P=0.9079	F (1, 36) = 1.519	P=0.2257	F (1, 36) = 0.04194	P=0.8389
2E	F (1, 44) = 4.188	P=0.0467	F (1, 44) = 4.820	P=0.0334	F (1, 44) = 152.2	P<0.0001
2F	F (1, 51) = 1.077	P=0.3042	F (1, 51) = 2.915	P=0.0938	F (1, 51) = 12.50	P=0.0009
3A	F (1, 24) = 0.1096	P=0.7435	F (1, 24) = 29.71	P<0.0001	F (1, 24) = 7.167	P=0.0132
3C	F (1, 18) = 0.1978	P=0.6618	F (1, 18) = 10.53	P=0.0045	F (1, 18) = 0.6761	P=0.4217
3D	F (1, 12) = 11.35	P=0.0056	F (1, 12) = 65.30	P<0.0001	F (1, 12) = 93.88	P<0.0001
3E	F (1, 12) = 18.84	P=0.0010	F (1, 12) = 3.821	P=0.0743	F (1, 12) = 4.085	P=0.0662
4A (left)	F (1, 12) = 8.184	P=0.0143	F (1, 12) = 9.481	P=0.0096	F (1, 12) = 20.54	P=0.0007
4A (right)	F (1, 20) = 6.489	P=0.0192	F (1, 20) = 10.72	P=0.0038	F (1, 20) = 5.881	P=0.0249
4B (left)	F (1, 12) = 38.48	P<0.0001	F (1, 12) = 46.69	P<0.0001	F (1, 12) = 34.77	P<0.0001
4B (right)	F (1, 12) = 11.97	P=0.0047	F (1, 12) = 13.60	P=0.0031	F (1, 12) = 1.506	P=0.2434
5A	F (1, 14) = 7.788	P=0.0144	F (1, 14) = 0.02021	P=0.8890	F (1, 14) = 0.07663	P=0.7860
5C (filopodium + thin)	F (1, 14) = 15.75	P=0.0014	F (1, 14) = 18.53	P=0.0007	F (1, 14) = 22.88	P=0.0003
5C (stubby)	F (1, 14) = 0.2823	P=0.6035	F (1, 14) = 1.010	P=0.3319	F (1, 14) = 5.664	P=0.0321
5C (mushroom)	F (1, 14) = 28.71	P=0.0001	F (1, 14) = 31.58	P<0.0001	F (1, 14) = 55.71	P<0.0001
5C (cup)	F (1, 14) = 0.1728	P=0.6840	F (1, 14) = 3.909	P=0.0681	F (1, 14) = 6.903	P=0.0199
5D	F (1, 14) = 67.07	P<0.0001	F (1, 14) = 149.0	P<0.0001	F (1, 14) = 262.9	P<0.0001
6B	F (1, 12) = 1.389	P=0.2614	F (1, 12) = 64.27	P<0.0001	F (1, 12) = 8.001	P=0.0152
6C	F (1, 47) = 0.4684	P=0.4971	F (1, 47) = 33.53	P<0.0001	F (1, 47) = 2.902	P=0.0951

Uncorrected Fisher's LSD

Figure 1C

Uncorrected Fisher's LSD	Mean Diff.	95.00% CI of diff.	Significant	Summary	Individual P Value
Runner *Cdkl5* +/Y vs. Runner *Cdkl5* -/Y					
Day 7	4844	193.4 to 9495	Yes	*	0.0422
Day 14	11271	716.6 to 21825	Yes	*	0.0386
Day 21	26271	12477 to 40065	Yes	**	0.0013
Day 28	15661	4884 to 26438	Yes	**	0.0069
Runner *Cdkl5* +/Y					
Day 7 vs. Day 14	-13452	-20608 to -6296	Yes	**	0.003
Day 7 vs. Day 21	-31110	-41818 to -20401	Yes	***	0.0002
Day 7 vs. Day 28	-21384	-29482 to -13287	Yes	***	0.0004
Day 14 vs. Day 21	-17658	-23930 to -11385	Yes	***	0.0003
Day 14 vs. Day 28	-7932	-15794 to -70.42	Yes	*	0.0485
Day 21 vs. Day 28	9726	-833.4 to 20285	No	ns	0.0658
Runner *Cdkl5* -/Y					
Day 7 vs. Day 14	-7026	-11131 to -2920	Yes	**	0.0031
Day 7 vs. Day 21	-9683	-17284 to -2082	Yes	*	0.0172
Day 7 vs. Day 28	-10567	-19219 to -1916	Yes	*	0.0211
Day 14 vs. Day 21	-2658	-8081 to 2765	No	ns	0.3038
Day 14 vs. Day 28	-3542	-9446 to 2362	No	ns	0.2135
Day 21 vs. Day 28	-884.2	-3320 to 1552	No	ns	0.4412

Figure 1D

Uncorrected Fisher's LSD	Predicted (LS) Mean Diff.	95.00% CI of diff.	Significant	Summary	Individual P Value
Day 1					
Sedentary *Cdkl5* +/Y vs. Sedentary *Cdkl5* -/Y	1.185	-1.515 to 3.886	No	ns	0.3878
Sedentary *Cdkl5* +/Y vs. Runner *Cdkl5* -/Y	0.05667	-2.578 to 2.691	No	ns	0.9662
Sedentary *Cdkl5* +/Y vs. Runner *Cdkl5* +/Y	0.69	-2.288 to 3.668	No	ns	0.6483
Sedentary *Cdkl5* -/Y vs. Runner *Cdkl5* -/Y	-1.129	-3.968 to 1.711	No	ns	0.4341
Sedentary *Cdkl5* -/Y vs. Runner *Cdkl5* +/Y	-0.4955	-3.656 to 2.665	No	ns	0.7576
Runner *Cdkl5* -/Y vs. Runner *Cdkl5* +/Y	0.6333	-2.472 to 3.738	No	ns	0.688

Day 8					
Sedentary *Cdkl5* +/Y vs. Sedentary *Cdkl5* -/Y	0.36	-2.340 to 3.060	No	ns	0.7929
Sedentary *Cdkl5* +/Y vs. Runner *Cdkl5* -/Y	-0.54	-3.175 to 2.095	No	ns	0.6866
Sedentary *Cdkl5* +/Y vs. Runner *Cdkl5* +/Y	0.3975	-2.581 to 3.376	No	ns	0.7927
Sedentary *Cdkl5* -/Y vs. Runner *Cdkl5* -/Y	-0.9	-3.739 to 1.939	No	ns	0.5328
Sedentary *Cdkl5* -/Y vs. Runner *Cdkl5* +/Y	0.0375	-3.123 to 3.198	No	ns	0.9814
Runner *Cdkl5* -/Y vs. Runner *Cdkl5* +/Y	0.9375	-2.167 to 4.042	No	ns	0.5523
Day 15					
Sedentary *Cdkl5* +/Y vs. Sedentary *Cdkl5* -/Y	0.02727	-2.673 to 2.728	No	ns	0.9841
Sedentary *Cdkl5* +/Y vs. Runner *Cdkl5* -/Y	-0.825	-3.460 to 1.810	No	ns	0.5377
Sedentary *Cdkl5* +/Y vs. Runner *Cdkl5* +/Y	0.275	-2.703 to 3.253	No	ns	0.8557
Sedentary *Cdkl5* -/Y vs. Runner *Cdkl5* -/Y	-0.8523	-3.692 to 1.987	No	ns	0.5547
Sedentary *Cdkl5* -/Y vs. Runner *Cdkl5* +/Y	0.2477	-2.913 to 3.409	No	ns	0.8774
Runner *Cdkl5* -/Y vs. Runner *Cdkl5* +/Y	1.1	-2.005 to 4.205	No	ns	0.4857
Day 22					
Sedentary *Cdkl5* +/Y vs. Sedentary *Cdkl5* -/Y	0.5158	-2.184 to 3.216	No	ns	0.7069
Sedentary *Cdkl5* +/Y vs. Runner *Cdkl5* -/Y	-0.6683	-3.303 to 1.966	No	ns	0.6175
Sedentary *Cdkl5* +/Y vs. Runner *Cdkl5* +/Y	0.1942	-2.784 to 3.172	No	ns	0.8979
Sedentary *Cdkl5* -/Y vs. Runner *Cdkl5* -/Y	-1.184	-4.024 to 1.655	No	ns	0.412
Sedentary *Cdkl5* -/Y vs. Runner *Cdkl5* +/Y	-0.3216	-3.482 to 2.839	No	ns	0.8412
Runner *Cdkl5* -/Y vs. Runner *Cdkl5* +/Y	0.8625	-2.242 to 3.967	No	ns	0.5845
Day 30					
Sedentary *Cdkl5* +/Y vs. Sedentary *Cdkl5* -/Y	0.3558	-2.344 to 3.056	No	ns	0.7953
Sedentary *Cdkl5* +/Y vs. Runner *Cdkl5* -/Y	-0.9117	-3.546 to 1.723	No	ns	0.4959
Sedentary *Cdkl5* +/Y vs. Runner *Cdkl5* +/Y	-0.1658	-3.144 to 2.812	No	ns	0.9127
Sedentary *Cdkl5* -/Y vs. Runner *Cdkl5* -/Y	-1.267	-4.107 to 1.572	No	ns	0.3799
Sedentary *Cdkl5* -/Y vs. Runner *Cdkl5* +/Y	-0.5216	-3.682 to 2.639	No	ns	0.7453
Runner *Cdkl5* -/Y vs. Runner *Cdkl5* +/Y	0.7458	-2.359 to 3.851	No	ns	0.6363

Figure 2A

Uncorrected Fisher's LSD	Mean Diff.	95.00% CI of diff.	Significant	Summary	Individual P Value
Sedentary *Cdkl5* +/Y vs. Sedentary *Cdkl5* -/Y	-1418	-2248 to -587.2	Yes	**	0.0014
Sedentary *Cdkl5* +/Y vs. Runner *Cdkl5* +/Y	546.1	-386.3 to 1478	No	ns	0.2427
Sedentary *Cdkl5* +/Y vs. Runner *Cdkl5* -/Y	-278	-1251 to 694.6	No	ns	0.5657

Sedentary *Cdkl5* -/Y vs. Runner *Cdkl5* +/Y	1964	1017 to 2911	Yes	***	0.0002
Sedentary *Cdkl5* -/Y vs. Runner *Cdkl5* -/Y	1140	152.9 to 2126	Yes	*	0.0248
Runner *Cdkl5* +/Y vs. Runner *Cdkl5* -/Y	-824.1	-1898 to 249.7	No	ns	0.1283

Figure 2B

Uncorrected Fisher's LSD	Mean Diff.	95.00% CI of diff.	Significant	Summary	Individual P Value
Sedentary *Cdkl5* +/Y vs. Sedentary *Cdkl5* -/Y	-2.832	-4.211 to -1.453	Yes	***	0.0002
Sedentary *Cdkl5* +/Y vs. Runner *Cdkl5* +/Y	1.101	-0.4469 to 2.649	No	ns	0.1578
Sedentary *Cdkl5* +/Y vs. Runner *Cdkl5* -/Y	-0.3443	-1.959 to 1.271	No	ns	0.668
Sedentary *Cdkl5* -/Y vs. Runner *Cdkl5* +/Y	3.933	2.361 to 5.505	Yes	****	<0.0001
Sedentary *Cdkl5* -/Y vs. Runner *Cdkl5* -/Y	2.488	0.8494 to 4.126	Yes	**	0.004
Runner *Cdkl5* +/Y vs. Runner *Cdkl5* -/Y	-1.445	-3.228 to 0.3374	No	ns	0.1088

Figure 2C

Uncorrected Fisher's LSD	Mean Diff.	95.00% CI of diff.	Significant	Summary	Individual P Value
Sedentary *Cdkl5* +/Y vs. Sedentary *Cdkl5* -/Y	-57.73	-91.38 to -24.09	Yes	**	0.0013
Sedentary *Cdkl5* +/Y vs. Runner *Cdkl5* +/Y	-15.23	-53.00 to 22.53	No	ns	0.4187
Sedentary *Cdkl5* +/Y vs. Runner *Cdkl5* -/Y	-7.919	-47.32 to 31.48	No	ns	0.686
Sedentary *Cdkl5* -/Y vs. Runner *Cdkl5* +/Y	42.5	4.137 to 80.86	Yes	*	0.0309
Sedentary *Cdkl5* -/Y vs. Runner *Cdkl5* -/Y	49.81	9.841 to 89.78	Yes	*	0.016
Runner *Cdkl5* +/Y vs. Runner *Cdkl5* -/Y	7.314	-36.18 to 50.81	No	ns	0.7351

Figure 2D

Uncorrected Fisher's LSD	Mean Diff.	95.00% CI of diff.	Significant	Summary	Individual P Value
Sedentary *Cdkl5* +/Y vs. Sedentary *Cdkl5* -/Y	-2.077	-60.45 to 56.29	No	ns	0.9429
Sedentary *Cdkl5* +/Y vs. Runner *Cdkl5* +/Y	-26.25	-91.77 to 39.27	No	ns	0.4218
Sedentary *Cdkl5* +/Y vs. Runner *Cdkl5* -/Y	-33.81	-102.2 to 34.55	No	ns	0.3225
Sedentary *Cdkl5* -/Y vs. Runner *Cdkl5* +/Y	-24.18	-90.73 to 42.38	No	ns	0.4661
Sedentary *Cdkl5* -/Y vs. Runner *Cdkl5* -/Y	-31.73	-101.1 to 37.61	No	ns	0.3596
Runner *Cdkl5* +/Y vs. Runner *Cdkl5* -/Y	-7.556	-83.02 to 67.91	No	ns	0.8402

Figure 2E

Uncorrected Fisher's LSD	Mean Diff.	95.00% CI of diff.	Significant	Summary	Individual P Value
Sedentary *Cdkl5* +/Y vs. Sedentary *Cdkl5* -/Y	-65.27	-81.30 to -49.25	Yes	****	<0.0001
Sedentary *Cdkl5* +/Y vs. Runner *Cdkl5* +/Y	-0.946	-19.60 to 17.71	No	ns	0.919
Sedentary *Cdkl5* +/Y vs. Runner *Cdkl5* -/Y	-92.17	-107.8 to -76.57	Yes	****	<0.0001

Sedentary *Cdkl5* -/Y vs. Runner *Cdkl5* +/Y	64.32	44.08 to 84.57	Yes	****	<0.0001
Sedentary *Cdkl5* -/Y vs. Runner *Cdkl5* -/Y	-26.9	-44.38 to -9.422	Yes	**	0.0034
Runner *Cdkl5* +/Y vs. Runner *Cdkl5* -/Y	-91.23	-111.1 to -71.31	Yes	****	<0.0001

Figure 2F

Uncorrected Fisher's LSD	Mean Diff.	95.00% CI of diff.	Significant	Summary	Individual P Value
Sedentary *Cdkl5* +/Y vs. Sedentary *Cdkl5* -/Y	168	88.41 to 247.5	Yes	****	<0.0001
Sedentary *Cdkl5* +/Y vs. Runner *Cdkl5* +/Y	-24.59	-131.5 to 82.32	No	ns	0.6463
Sedentary *Cdkl5* +/Y vs. Runner *Cdkl5* -/Y	67.14	-30.33 to 164.6	No	ns	0.1727
Sedentary *Cdkl5* -/Y vs. Runner *Cdkl5* +/Y	-192.6	-303.2 to -81.92	Yes	***	0.001
Sedentary *Cdkl5* -/Y vs. Runner *Cdkl5* -/Y	-100.8	-202.4 to 0.7349	No	ns	0.0516
Runner *Cdkl5* +/Y vs. Runner *Cdkl5* -/Y	91.73	-32.42 to 215.9	No	ns	0.1441

Figure 3A

Uncorrected Fisher's LSD	Mean Diff.	95.00% CI of diff.	Significant	Summary	Individual P Value
Sedentary *Cdkl5* +/Y vs. Sedentary *Cdkl5* -/Y	0.7363	-0.2430 to 1.716	No	ns	0.1338
Sedentary *Cdkl5* +/Y vs. Runner *Cdkl5* +/Y	-1.815	-2.731 to -0.8987	Yes	***	0.0004
Sedentary *Cdkl5* +/Y vs. Runner *Cdkl5* -/Y	-0.8706	-1.787 to 0.04545	No	ns	0.0615
Sedentary *Cdkl5* -/Y vs. Runner *Cdkl5* +/Y	-2.551	-3.467 to -1.635	Yes	****	<0.0001
Sedentary *Cdkl5* -/Y vs. Runner *Cdkl5* -/Y	-1.607	-2.523 to -0.6909	Yes	**	0.0014
Runner *Cdkl5* +/Y vs. Runner *Cdkl5* -/Y	0.9441	0.09600 to 1.792	Yes	*	0.0306

Figure 3C

Uncorrected Fisher's LSD	Mean Diff.	95.00% CI of diff.	Significant	Summary	Individual P Value
Sedentary *Cdkl5* +/Y vs. Sedentary *Cdkl5* -/Y	0.01149	-0.09016 to 0.1131	No	ns	0.8149
Sedentary *Cdkl5* +/Y vs. Runner *Cdkl5* +/Y	-0.1123	-0.2051 to -0.01951	Yes	*	0.0204
Sedentary *Cdkl5* +/Y vs. Runner *Cdkl5* -/Y	-0.07375	-0.1618 to 0.01429	No	ns	0.0954
Sedentary *Cdkl5* -/Y vs. Runner *Cdkl5* +/Y	-0.1238	-0.2166 to -0.03101	Yes	*	0.0118
Sedentary *Cdkl5* -/Y vs. Runner *Cdkl5* -/Y	-0.08524	-0.1733 to 0.002800	No	ns	0.0569
Runner *Cdkl5* +/Y vs. Runner *Cdkl5* -/Y	0.03857	-0.03907 to 0.1162	No	ns	0.3105

Figure 3D

Uncorrected Fisher's LSD	Mean Diff.	95.00% CI of diff.	Significant	Summary	Individual P Value
Sedentary *Cdkl5* +/Y vs. Sedentary *Cdkl5* -/Y	0.8745	0.6682 to 1.081	Yes	****	<0.0001
Sedentary *Cdkl5* +/Y vs. Runner *Cdkl5* +/Y	-0.3155	-0.5219 to -0.1092	Yes	**	0.006
Sedentary *Cdkl5* +/Y vs. Runner *Cdkl5* -/Y	0.1077	-0.09866 to 0.3140	No	ns	0.2777

Sedentary *Cdkl5* -/Y vs. Runner *Cdkl5* +/Y	-1.19	-1.396 to -0.9837	Yes	****	<0.0001
Sedentary *Cdkl5* -/Y vs. Runner *Cdkl5* -/Y	-0.7668	-0.9732 to -0.5605	Yes	****	<0.0001
Runner *Cdkl5* +/Y vs. Runner *Cdkl5* -/Y	0.4232	0.2169 to 0.6296	Yes	***	0.0008

Figure 3E

Uncorrected Fisher's LSD	Mean Diff.	95.00% CI of diff.	Significant	Summary	Individual P Value
Sedentary *Cdkl5* +/Y vs. Sedentary *Cdkl5* -/Y	0.24	0.1237 to 0.3563	Yes	***	0.0007
Sedentary *Cdkl5* +/Y vs. Runner *Cdkl5* +/Y	0.09	-0.02625 to 0.2063	No	ns	0.1174
Sedentary *Cdkl5* +/Y vs. Runner *Cdkl5* -/Y	0.0025	-0.1138 to 0.1188	No	ns	0.9634
Sedentary *Cdkl5* -/Y vs. Runner *Cdkl5* +/Y	-0.15	-0.2663 to -0.03375	Yes	*	0.0157
Sedentary *Cdkl5* -/Y vs. Runner *Cdkl5* -/Y	-0.2375	-0.3538 to -0.1212	Yes	***	0.0008
Runner *Cdkl5* +/Y vs. Runner *Cdkl5* -/Y	-0.0875	-0.2038 to 0.02875	No	ns	0.127

Figure 4A (left)

Uncorrected Fisher's LSD	Mean Diff.	95.00% CI of diff.	Significant	Summary	Individual P Value
Sedentary *Cdkl5* +/Y vs. Sedentary *Cdkl5* -/Y	-6.348	-8.993 to -3.702	Yes	***	0.0002
Sedentary *Cdkl5* +/Y vs. Runner *Cdkl5* +/Y	0.1875	-2.458 to 2.833	No	ns	0.8798
Sedentary *Cdkl5* +/Y vs. Runner *Cdkl5* -/Y	-1.248	-3.893 to 1.398	No	ns	0.3245
Sedentary *Cdkl5* -/Y vs. Runner *Cdkl5* +/Y	6.535	3.889 to 9.181	Yes	***	0.0002
Sedentary *Cdkl5* -/Y vs. Runner *Cdkl5* -/Y	5.1	2.454 to 7.746	Yes	**	0.0012
Runner *Cdkl5* +/Y vs. Runner *Cdkl5* -/Y	-1.435	-4.081 to 1.211	No	ns	0.2602

Figure 4A (right)

Uncorrected Fisher's LSD	Mean Diff.	95.00% CI of diff.	Significant	Summary	Individual P Value
Sedentary *Cdkl5* +/Y vs. Sedentary *Cdkl5* -/Y	-1.286	-1.909 to -0.6630	Yes	***	0.0003
Sedentary *Cdkl5* +/Y vs. Runner *Cdkl5* +/Y	0.1881	-0.5748 to 0.9510	No	ns	0.6126
Sedentary *Cdkl5* +/Y vs. Runner *Cdkl5* -/Y	0.2198	-0.5431 to 0.9827	No	ns	0.5546
Sedentary *Cdkl5* -/Y vs. Runner *Cdkl5* +/Y	1.474	0.7111 to 2.237	Yes	***	0.0007
Sedentary *Cdkl5* -/Y vs. Runner *Cdkl5* -/Y	1.506	0.7428 to 2.269	Yes	***	0.0005
Runner *Cdkl5* +/Y vs. Runner *Cdkl5* -/Y	0.03166	-0.8492 to 0.9126	No	ns	0.941

Figure 4B (left)

Uncorrected Fisher's LSD	Mean Diff.	95.00% CI of diff.	Significant	Summary	Individual P Value
Sedentary *Cdkl5* +/Y vs. Sedentary *Cdkl5* -/Y	-7.593	-9.526 to -5.659	Yes	****	<0.0001
Sedentary *Cdkl5* +/Y vs. Runner *Cdkl5* +/Y	0.395	-1.538 to 2.328	No	ns	0.6641
Sedentary *Cdkl5* +/Y vs. Runner *Cdkl5* -/Y	0.5875	-1.346 to 2.521	No	ns	0.5204

Sedentary *Cdkl5* -/Y vs. Runner *Cdkl5* +/Y	7.988	6.054 to 9.921	Yes	****	<0.0001
Sedentary *Cdkl5* -/Y vs. Runner *Cdkl5* -/Y	8.18	6.247 to 10.11	Yes	****	<0.0001
Runner *Cdkl5* +/Y vs. Runner *Cdkl5* -/Y	0.1925	-1.741 to 2.126	No	ns	0.8319

Figure 4B (right)

Uncorrected Fisher's LSD	Mean Diff.	95.00% CI of diff.	Significant	Summary	Individual P Value
Sedentary *Cdkl5* +/Y vs. Sedentary *Cdkl5* -/Y	-0.7583	-1.257 to -0.2598	Yes	**	0.0062
Sedentary *Cdkl5* +/Y vs. Runner *Cdkl5* +/Y	0.03694	-0.4616 to 0.5355	No	ns	0.8744
Sedentary *Cdkl5* +/Y vs. Runner *Cdkl5* -/Y	0.3982	-0.1004 to 0.8967	No	ns	0.1074
Sedentary *Cdkl5* -/Y vs. Runner *Cdkl5* +/Y	0.7953	0.2967 to 1.294	Yes	**	0.0046
Sedentary *Cdkl5* -/Y vs. Runner *Cdkl5* -/Y	1.157	0.6579 to 1.655	Yes	***	0.0003
Runner *Cdkl5* +/Y vs. Runner *Cdkl5* -/Y	0.3613	-0.1373 to 0.8598	No	ns	0.1404

Figure 5A

Uncorrected Fisher's LSD	Mean Diff.	95.00% CI of diff.	Significant	Summary	Individual P Value
Sedentary *Cdkl5* +/Y vs. Sedentary *Cdkl5* -/Y	0.084	-0.01156 to 0.1796	No	ns	0.0803
Sedentary *Cdkl5* +/Y vs. Runner *Cdkl5* +/Y	0.0885	-0.01285 to 0.1899	No	ns	0.0821
Sedentary *Cdkl5* +/Y vs. Runner *Cdkl5* -/Y	-0.014	-0.1154 to 0.08735	No	ns	0.7714
Sedentary *Cdkl5* -/Y vs. Runner *Cdkl5* +/Y	0.0045	-0.09685 to 0.1059	No	ns	0.9255
Sedentary *Cdkl5* -/Y vs. Runner *Cdkl5* -/Y	-0.098	-0.1994 to 0.003354	No	ns	0.057
Runner *Cdkl5* +/Y vs. Runner *Cdkl5* -/Y	-0.1025	-0.2093 to 0.004336	No	ns	0.0587

Figure 5C (filopodium + thin)

Uncorrected Fisher's LSD	Mean Diff.	95.00% CI of diff.	Significant	Summary	Individual P Value
Sedentary *Cdkl5* +/Y vs. Sedentary *Cdkl5* -/Y	-14.26	-18.92 to -9.599	Yes	****	<0.0001
Sedentary *Cdkl5* +/Y vs. Runner *Cdkl5* +/Y	0.548	-4.393 to 5.489	No	ns	0.8154
Sedentary *Cdkl5* +/Y vs. Runner *Cdkl5* -/Y	-0.7795	-5.721 to 4.162	No	ns	0.7401
Sedentary *Cdkl5* -/Y vs. Runner *Cdkl5* +/Y	14.81	9.865 to 19.75	Yes	****	<0.0001
Sedentary *Cdkl5* -/Y vs. Runner *Cdkl5* -/Y	13.48	8.537 to 18.42	Yes	****	<0.0001
Runner *Cdkl5* +/Y vs. Runner *Cdkl5* -/Y	-1.328	-6.536 to 3.881	No	ns	0.5932

Figure 5C (stubby)

Uncorrected Fisher's LSD	Mean Diff.	95.00% CI of diff.	Significant	Summary	Individual P Value
Sedentary *Cdkl5* +/Y vs. Sedentary *Cdkl5* -/Y	-2.694	-6.861 to 1.473	No	ns	0.1873
Sedentary *Cdkl5* +/Y vs. Runner *Cdkl5* +/Y	2.239	-2.181 to 6.659	No	ns	0.2957
Sedentary *Cdkl5* +/Y vs. Runner *Cdkl5* -/Y	-2.004	-6.424 to 2.417	No	ns	0.3475

Sedentary *Cdkl5* -/Y vs. Runner *Cdkl5* +/Y	4.933	0.5127 to 9.353	Yes	*	0.0313
Sedentary *Cdkl5* -/Y vs. Runner *Cdkl5* -/Y	0.6905	-3.730 to 5.111	No	ns	0.7426
Runner *Cdkl5* +/Y vs. Runner *Cdkl5* -/Y	-4.243	-8.902 to 0.4169	No	ns	0.0711

Figure 5C (mushroom)

Uncorrected Fisher's LSD	Mean Diff.	95.00% CI of diff.	Significant	Summary	Individual P Value
Sedentary *Cdkl5* +/Y vs. Sedentary *Cdkl5* -/Y	14.64	11.38 to 17.91	Yes	****	<0.0001
Sedentary *Cdkl5* +/Y vs. Runner *Cdkl5* +/Y	-0.2985	-3.763 to 3.166	No	ns	0.856
Sedentary *Cdkl5* +/Y vs. Runner *Cdkl5* -/Y	2.106	-1.358 to 5.571	No	ns	0.2132
Sedentary *Cdkl5* -/Y vs. Runner *Cdkl5* +/Y	-14.94	-18.41 to -11.48	Yes	****	<0.0001
Sedentary *Cdkl5* -/Y vs. Runner *Cdkl5* -/Y	-12.54	-16.00 to -9.073	Yes	****	<0.0001
Runner *Cdkl5* +/Y vs. Runner *Cdkl5* -/Y	2.405	-1.247 to 6.057	No	ns	0.1796

Figure 5C (cup)

Uncorrected Fisher's LSD	Mean Diff.	95.00% CI of diff.	Significant	Summary	Individual P Value
Sedentary *Cdkl5* +/Y vs. Sedentary *Cdkl5* -/Y	2.304	-0.6750 to 5.283	No	ns	0.1194
Sedentary *Cdkl5* +/Y vs. Runner *Cdkl5* +/Y	-2.493	-5.652 to 0.6672	No	ns	0.1128
Sedentary *Cdkl5* +/Y vs. Runner *Cdkl5* -/Y	0.6775	-2.482 to 3.837	No	ns	0.6527
Sedentary *Cdkl5* -/Y vs. Runner *Cdkl5* +/Y	-4.797	-7.956 to -1.637	Yes	**	0.0057
Sedentary *Cdkl5* -/Y vs. Runner *Cdkl5* -/Y	-1.627	-4.786 to 1.533	No	ns	0.2882
Runner *Cdkl5* +/Y vs. Runner *Cdkl5* -/Y	3.17	-0.1606 to 6.501	No	ns	0.0605

Figure 5D

Uncorrected Fisher's LSD	Mean Diff.	95.00% CI of diff.	Significant	Summary	Individual P Value
Sedentary *Cdkl5* +/Y vs. Sedentary *Cdkl5* -/Y	16.95	14.96 to 18.93	Yes	****	<0.0001
Sedentary *Cdkl5* +/Y vs. Runner *Cdkl5* +/Y	-2.789	-4.896 to -0.6824	Yes	*	0.0131
Sedentary *Cdkl5* +/Y vs. Runner *Cdkl5* -/Y	2.784	0.6769 to 4.890	Yes	*	0.0133
Sedentary *Cdkl5* -/Y vs. Runner *Cdkl5* +/Y	-19.74	-21.84 to -17.63	Yes	****	<0.0001
Sedentary *Cdkl5* -/Y vs. Runner *Cdkl5* -/Y	-14.16	-16.27 to -12.06	Yes	****	<0.0001
Runner *Cdkl5* +/Y vs. Runner *Cdkl5* -/Y	5.573	3.352 to 7.793	Yes	****	<0.0001

Figure 6B

Uncorrected Fisher's LSD	Mean Diff.	95.00% CI of diff.	Significant	Summary	Individual P Value
Sedentary *Cdkl5* +/Y vs. Sedentary *Cdkl5* -/Y	-6.041	-17.32 to 5.241	No	ns	0.266
Sedentary *Cdkl5* +/Y vs. Runner *Cdkl5* +/Y	-25.04	-36.32 to -13.76	Yes	***	0.0004
Sedentary *Cdkl5* +/Y vs. Runner *Cdkl5* -/Y	-39.71	-50.99 to -28.43	Yes	****	<0.0001

Sedentary *Cdkl5* -/Y vs. Runner *Cdkl5* +/Y	-19	-30.28 to -7.714	Yes	**	0.0032
Sedentary *Cdkl5* -/Y vs. Runner *Cdkl5* -/Y	-33.67	-44.95 to -22.39	Yes	****	<0.0001
Runner *Cdkl5* +/Y vs. Runner *Cdkl5* -/Y	-14.67	-25.96 to -3.390	Yes	*	0.0151

Figure 6C

Uncorrected Fisher's LSD	Mean Diff.	95.00% CI of diff.	Significant	Summary	Individual P Value
Sedentary *Cdkl5* +/Y vs. Sedentary *Cdkl5* -/Y	-39.78	-81.24 to 1.684	No	ns	0.0596
Sedentary *Cdkl5* +/Y vs. Runner *Cdkl5* +/Y	-107.8	-158.3 to -57.34	Yes	****	<0.0001
Sedentary *Cdkl5* +/Y vs. Runner *Cdkl5* -/Y	-124.8	-169.5 to -80.14	Yes	****	<0.0001
Sedentary *Cdkl5* -/Y vs. Runner *Cdkl5* +/Y	-68.07	-118.0 to -18.11	Yes	**	0.0086
Sedentary *Cdkl5* -/Y vs. Runner *Cdkl5* -/Y	-85.04	-129.1 to -40.99	Yes	***	0.0003
Runner *Cdkl5* +/Y vs. Runner *Cdkl5* -/Y	-16.98	-69.63 to 35.68	No	ns	0.5197

Table S3. Descriptive statistics.
